# Innate-like NKp30^+^CD8^+^ T cells armed with TCR/CAR target tumor heterogeneity

**DOI:** 10.1080/2162402X.2021.1973783

**Published:** 2021-10-19

**Authors:** Margareta P. Correia, Ana Stojanovic, Winfried S. Wels, Adelheid Cerwenka

**Affiliations:** a Department of Immunobiochemistry, Mannheim Institute for Innate Immunosciences (MI3), Medical Faculty Mannheim, Heidelberg University, Mannheim, Germany; b Cancer Biology and Epigenetics Group, Research Center of IPO Porto (CI-IPOP)/RISE@CI-IPOP (Health Research Network), Portuguese Oncology Institute of Porto (IPO Porto)/Porto Comprehensive Cancer Center (Porto.CCC), Porto, Portugal; c Department of Pathology and Molecular Immunology, School of Medicine & Biomedical Sciences, University of Porto (ICBAS-UP), Porto, Portugal; d Georg-Speyer-Haus, Institute for Tumor Biology and Experimental Therapy, Frankfurt, Germany; e Frankfurt Cancer Institute, Goethe University, Frankfurt, Germany; f German Cancer Research Center (DKFZ), Heidelberg, Germany; g German Cancer Consortium (DKTK), Partner Site Frankfurt/Mainz, Frankfurt, Germany; h European Center for Angioscience (ECAS), Medical Faculty Mannheim, Heidelberg University, Mannheim, Germany

**Keywords:** CD8^+^ T cells, NKp30, innate T cells, CAR T cells, TCR-transduced T cells, immunotherapy

## Abstract

Intratumoral heterogeneity is frequently associated with tumor immune escape, with MHC-class I and antigen expression loss rendering tumor cells invisible to T cell killing, representing a major challenge for the design of successful adoptive transfer protocols for cancer immunotherapy. While CD8^+^ T cell recognition of tumor cells is based on the detection of MHC-peptide complexes via specific T cell receptors (TCRs), Natural Killer (NK) cells detect tumor-associated NK ligands by an array of NK receptors. We have recently identified a population of innate-like CD8^+^ T cells marked by the expression of NKp30, a potent natural cytotoxicity activating NK receptor, whose tumor ligand, B7H6, is frequently upregulated on several cancer types. Here, we harnessed the dual-recognition potential of NKp30^+^CD8^+^ T cells, by arming these cells with TCRs or chimeric antigen receptors (CARs) targeting Epidermal Growth Factor Receptor 2 (ErbB2, or HER2), a tumor-associated target overexpressed in several malignancies. HER2-specific NKp30^+^CD8^+^ T cells killed not only HER2-expressing target cell lines, but also eliminated tumor cells in the absence of MHC-class I or antigen expression, making them especially effective in eliminating heterogeneous tumor cell populations. Our results show that NKp30^+^CD8^+^ T cells equipped with a specific TCR or CAR display a dual capacity to recognize and kill target cells, combining the anti-tumor activity of both CD8^+^ T and NK cells. This dual-recognition capacity allows these effector cells to target tumor heterogeneity, thus improving therapeutic strategies against tumor escape.

## Introduction

Tumor escape is one of the major impediments for successful anti-tumor immune responses. Tumor heterogeneity is a key factor associated with tumor immune escape, occurring both among different patients (intertumoral heterogeneity), and among the cells composing the tumor tissue (intratumoral heterogeneity).^1^ Cancer heterogeneity increases upon therapy ultimately leading to the emergence of resistance, representing a serious challenge for targeted anti-tumor therapies.^1,2^ Additionally, MHC-class I downregulation and loss of antigen expression are classical tumor escape strategies, rendering tumor cells invisible to T cell recognition.^2^ Besides CD8^+^ T cells, Natural Killer (NK) cells are important anti-tumor effector cells. The recognition of tumor targets by NK cells does not rely on the MHC-peptide presentation, but rather on the induction of stress- and transformation-induced ligands on the surface of cancer cells, which often coincides with downregulation of MHC-class I molecules. Thus, NK cell activation is dictated by the balance between activating and inhibitory signals integrated upon ligand recognition by an array of receptors at the NK cell surface.^3,4^ Within the activating NK receptors, the family of natural cytotoxicity receptors (NCRs) comprises NKp30, NKp46 and NKp44. Although these receptors were already discovered in the 1990s,^5–7^ their ligands expressed on tumor cells are yet not fully characterized, with the exception of the tumor-associated NKp30 ligand, B7H6.^8^ Indeed, B7H6 was shown to be overexpressed on several cancer types, while rarely found on normal tissues under steady-state conditions,^9–13^ rendering this receptor/ligand axis attractive for anti-tumor immunotherapy.

The adoptive transfer of genetically-modified anti-tumor CD8^+^ T cells, equipped with tumor-reactive T cell receptors (TCRs) or chimeric antigen receptors (CARs), is an emerging strategy of personalized cancer immunotherapy.^14,15^ However, tumor heterogeneity frequently leads to escape from the recognition by CD8^+^ T cells, representing a major obstacle for successful cell-based immunotherapies.^2,16^ It has been known for some time that human CD8^+^ T cells express NKG2D, and that a small percentage of CD8^+^ T cells can express other NK cell receptors, such as KIRs, NKG2A and NKp46, under certain conditions.^17–22^ However, we have recently uncovered the existence of a distinct population of innate-like CD8^+^ T cells displaying broad NK features, marked by the expression of NKp30, a potent activating natural cytotoxicity receptor, which was until recently considered to be truly specific of NK cells. Moreover, we identified IL-15 as a signal capable of driving the *de novo* acquisition of NKp30 and initiating a CD8^+^ T cell reprogramming toward innate.^23^ This cell population endowed with both the CD8^+^ T cell and NK cell tumor recognition principle, could hold promise for immunotherapeutic approaches against cancer.

The human epidermal growth factor receptor (HER)2, or ErbB2, is a transmembrane receptor protein with an intracellular tyrosine kinase domain, and member of the epidermal growth factor receptor (EGFR) family. With no known ligand to date, HER2 can be activated by heterodimerization with other members of the EGFR family or by homodimerization when overexpressed on the cell surface.^24,25^ HER2 overexpression has been described in a wide variety of cancers, including breast, ovarian, colon, bladder, gastric, uterine cervix, head and neck, esophageal cancer, melanoma, and non-small cell lung cancer,^24,26,27,28^ making HER2 a well-established therapeutic tumor target.

Here, we aimed at harnessing the NKp30^+^CD8^+^ T cell population with tumor-reactive TCRs or CARs, combining the innate and adaptive characteristics of this population by introducing tumor-targeted specificity. We successfully armed innate-like NKp30^+^CD8^+^ T cells with tumor-reactive TCRs or CARs targeting HER2, thus endowing this CD8^+^ T cell population with dual-recognition potential against tumors, both in an MHC/antigen-dependent and independent manner. Our data indicate that NKp30^+^CD8^+^ T cells can be exploited for their anti-tumor activity, that combines both recognition capabilities of CD8^+^ T cells and NK cells, broadening the spectrum of tumor cells recognized and eliminated by adoptively transferred effector cells.

## Results

### Heterogeneous expression of HER2 and B7H6 in tumor tissues

The HER2 proto-oncogene is widely appreciated as a therapeutic tumor target due to its overexpression in various solid tumors. Similarly, the cognate tumor-associated ligand of NKp30, B7H6, was found to be upregulated on the cell surface of several tumor cell lines and in tumor tissues.^8–13^

**Figure 1.**
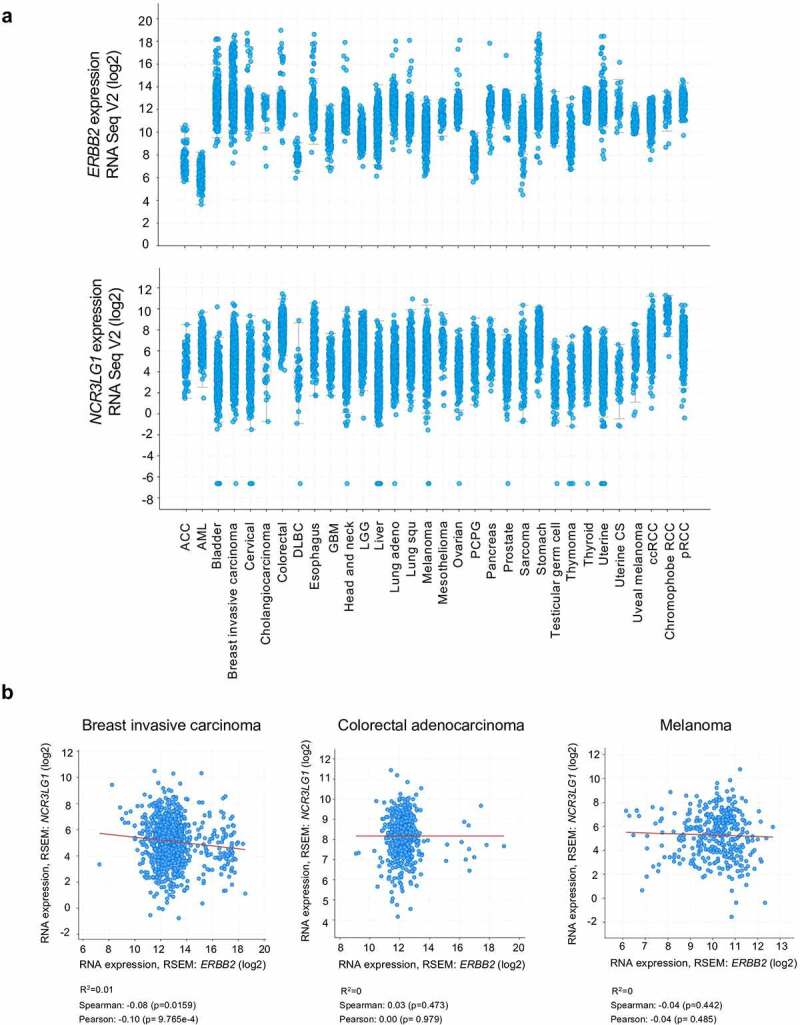
Expression of B7H6 and HER2 transcripts in different cancer types. (a) Expression data of *ERBB2* and *NCR3LG1* transcripts from different tumor types from TCGA datasets were analyzed and extracted from the cBioPortal for Cancer Genomics (https://cbioportal.org). (b) Correlation of *ERBB2* and *NCR3LG1* expression in individual patients with invasive breast carcinoma, colorectal adenocarcinoma and melanoma (spearman and Pearson correlative coefficients are shown). Graphs were generated and correlations were analyzed using the cBioPortal software

In silico analysis of transcriptional data from TCGA datasets (PanCancer Atlas) derived from cancer patients shows that HER2 (*ERBB2*) and B7H6 (*NCR3LG1*) transcripts are expressed in different tumor entities (Figure 1(a)). By analyzing selected tumor types, as invasive breast cancer, colorectal adenocarcinoma and skin cutaneous melanoma, we observed that both *ERBB2* and *NCR3LG1* transcripts show a variable, heterogeneous and non-correlative expression for each individual patient (Figure 1(b)). Thus, HER2 and B7H6 display variable expression, not only within different types of tumors, but also within the same tumor type across individual patients.

**Figure 2.**
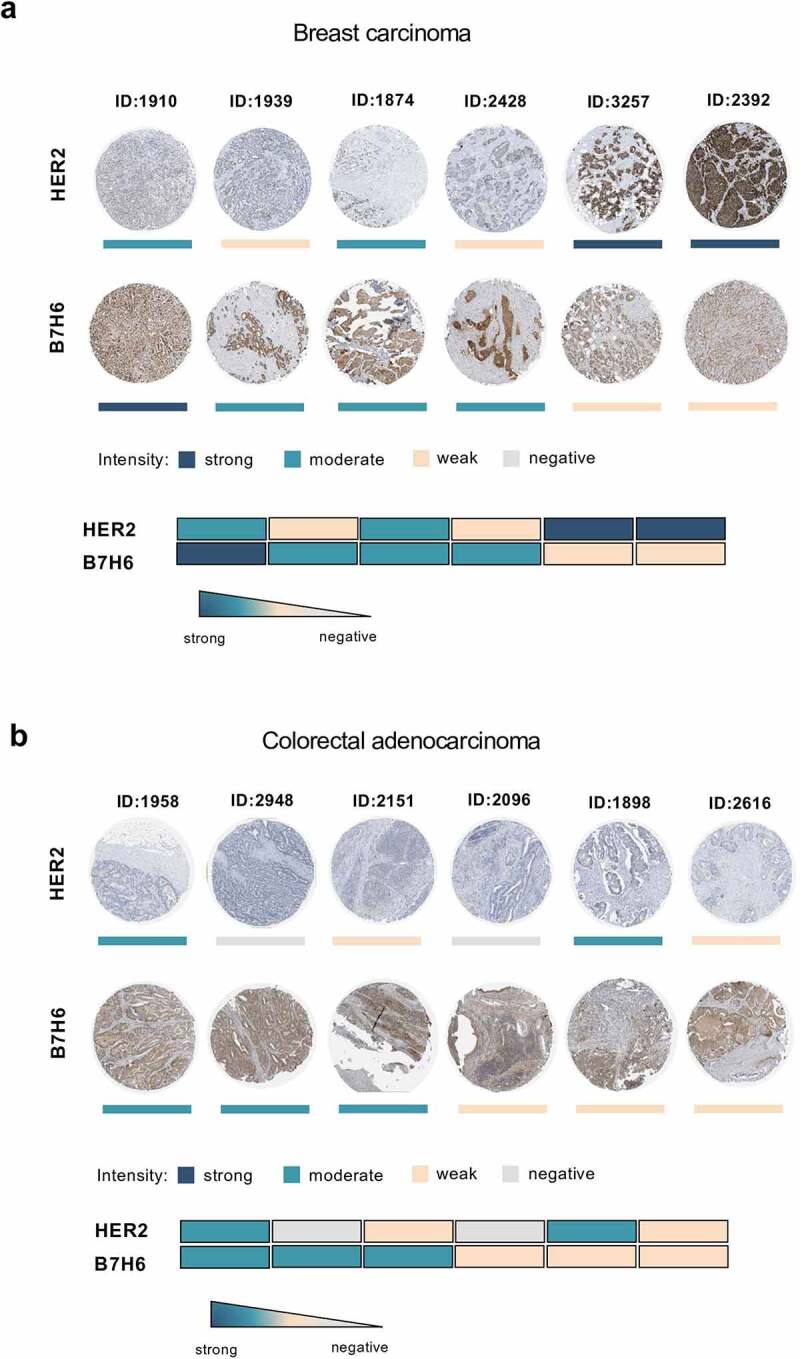
Heterogeneous B7H6 and HER2 protein expression in tumor samples from breast cancer and colon adenocarcinoma patients. (a-b) Immunohistochemistry slides of paired HER2 and B7H6 protein expression for (a) breast cancer and (b) colon cancer tumor samples. Color bars indicate staining intensity. Images were originally obtained from the Human Protein Atlas database (http://www.proteinatlas.org/). Heatmaps showing variability of HER2 and B7H6 expression for each sample. HER2 was stained with a mouse anti-human HER2 antibody (Leica Biosystems, Cat#NCL-CBE-356, RRID:AB_442047) and B7H6 with a polyclonal rabbit anti-human B7H6 antibody (Atlas Antibodies, Cat#HPA024137, RRID:AB_1859534, Sigma-Aldrich)

Staining of HER2 and B7H6 protein in breast carcinoma and colorectal adenocarcinoma samples (Human Protein Atlas database) shows protein expression of both molecules in different tumors and reveals a heterogeneous intratumoral expression (Figure 2(a-b)). We observed that some tumor samples displayed high expression of B7H6 and low expression of HER2 (e.g. breast carcinoma #1910 and colorectal adenocarcinoma #2948), while other samples displayed high HER2 and low B7H6 expression (e.g. breast carcinoma #2392 and colorectal adenocarcinoma #1898) (Figure 2(a-b)). Moreover, for both HER2 and B7H6, we observed intratumoral heterogeneity within each tumor sample, with not all cells composing a HER2^+^ or B7H6^+^ tumor sample expressing HER2 or B7H6, respectively (Figure 2(a-b)). Due to the observed inherent inter- and intratumoral heterogeneity within the tumor tissues, the ability to target more than one tumor-specific molecule by the same immune effector cells appears highly relevant.

### ^HER*2*^TCR^+^NKp30^+^CD8^+^ T cells exhibit a dual response based on both HER2 and B7H6 recognition

**Figure 3.**
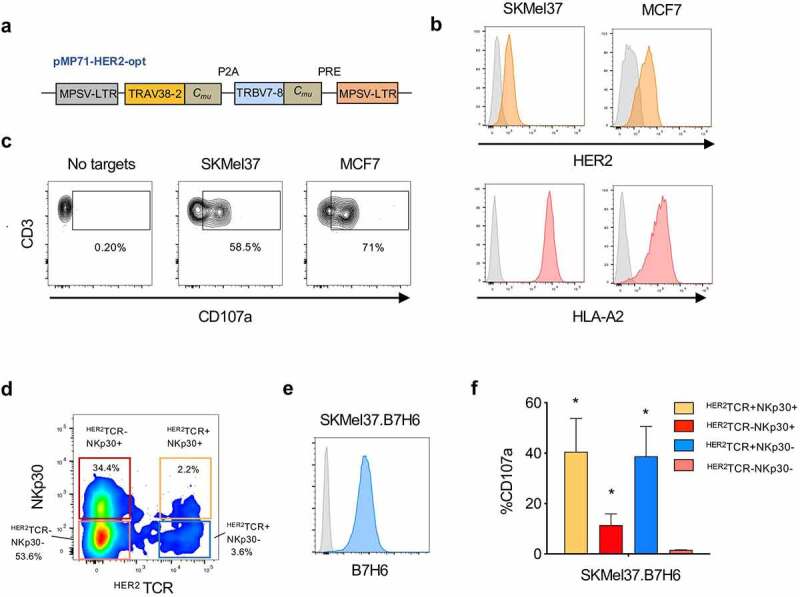
^HER2^TCR^+^CD8^+^ T cells recognize and respond to tumor cells expressing HER2. CD8^+^ T cells were expanded in the presence of αCD3/CD28 and IL-15 for 3 days, transduced with retroviral particles encoding the ^HER2^TCR and then cultured with IL-15 for 12 more days. (a) Simplified schematic representation of the ^HER2^TCR construct. (b) Cell lines were selected according to HER2 and HLA-A2 expression on the cell surface (isotype control in gray). SKMel37 (melanoma cell line), MCF7 (breast cancer cell line). (c) Dot-plot showing degranulation after co-culture with HER2-expressing target cells. (d) Dot-plot showing gating of the four CD8^+^ T cell subpopulations according to NKp30 and ectopic ^HER2^TCR expression before FACS-sorting for functional assays. (e) Histogram showing expression of B7H6 on SKMel37.B7H6 tumor cells (isotype control in gray). (f) Graph showing degranulation (CD107a^+^) on gated ^HER2^TCR^+^NKp30^+^, ^HER2^TCR^−^NKp30^+^, ^HER2^TCR^+^NKp30^−^ or ^HER2^TCR^−^NKp30^−^ T cells after co-culture with SKMel37.B7H6 cells for 4 h in the presence of anti-CD107a antibodies. n ≥ 3 independent experiments, mean±SEM. *P < .05, unpaired students *t*-test (comparison with the ^HER2^TCR^−^NKp30^−^ T cell population)

In a recent study, we uncovered a population of CD8^+^ T cells expressing NKp30 and displaying increased anti-tumor NK-like effector potential (Figure S1). Moreover, we found that IL-15 can *de novo* induce the coordinated expression of NKp30 and its adaptor molecule FcεRIγ on CD8^+^ T cells, leading to the generation and expansion of functional NKp30^+^CD8^+^ T cells.^23^ Here, we aimed at arming those NKp30^+^CD8^+^ T cells with a tumor-specific TCR targeting HER2, to direct CD8^+^ T cell responses directly to the tumor. To this end, we used a retroviral vector encoding a HER2-specific TCR restricted to HLA-A2, composed of murinized and codon-optimized P2A-linked TCRα- and TCRβ-chains^29^ (Figure 3(a)). We successfully transduced CD8^+^ T cells with the HER2-specific TCR (^HER2^TCR) (Figure S2a). Importantly, we were able to generate CD8^+^ T cells concomitantly expressing NKp30 and harboring a specific ^HER2^TCR: ^HER2^TCR^+^NKp30^+^CD8^+^ T cells (Figure 3(d) and Figure S2b).

Next, we assessed the functional response of ^HER2^TCR^+^CD8^+^ T cells against different tumor targets expressing HER2. We selected tumor cell lines expressing HLA-A2 at the cell surface in order to allow specific recognition by the transduced TCR (Figure 3(b)). Co-culture of ^HER2^TCR^+^CD8^+^ T cells with HER2-expressing tumor cell lines, resulted in the degranulation of ^HER2^TCR-transduced T cells (Figure 3(c)), showing that the generated CD8^+^ T cells carrying the specific TCR were functional.

Subsequently, we investigated whether ^HER2^TCR^+^NKp30^+^CD8^+^ T cells could display dual recognition toward target cells expressing both HER2 and B7H6. We evaluated the functional responses of all four paired subpopulations from each donor: NKp30^+^CD8^+^ T cell population, bearing or not HER2-specific TCRs (^HER2^TCR^+^NKp30^+^, ^HER2^TCR^−^NKp30^+^), and conventional NKp30^−^CD8^+^ T cells transduced or not with HER2-specific TCRs (^HER2^TCR^+^NKp30^−^, ^HER2^TCR^−^NKp30^−^) (Figure 3(d)). As tumor targets, we used an HER2^+^ cell line overexpressing B7H6, SKMel37.B7H6 (Figure 3(e)). CD8^+^ T cells bearing the ectopically expressed HER2-specific TCR (^HER2^TCR^+^NKp30^−^) degranulated in response to the HER2-expressing cell line, while ^HER2^TCR^−^NKp30^−^CD8^+^ T cells could not, indicating specificity of response (Figure 3(f)). Moreover, ^HER2^TCR^−^NKp30^+^CD8^+^ T cells degranulated in response to the tumor cell line, in a TCR-independent manner (Figure 3(f)), as we have previously shown.^23^ Importantly, the introduction of the HER2-specific TCR (^HER2^TCR^+^NKp30^+^) markedly increased the response of NKp30^+^CD8^+^ T cells (Figure 3(f)). These data show that ^HER2^TCR-bearing CD8^+^ T cells can effectively recognize HER2-expressing targets, with ^HER2^TCR^+^NKp30^+^CD8^+^ T cells being able to recognize and respond in both a TCR-dependent and independent NK-like manner.

### ^HER2^TCR^+^NKp30^+^CD8^+^ T cells recognize and kill MHC-class I loss variants

**Figure 4.**
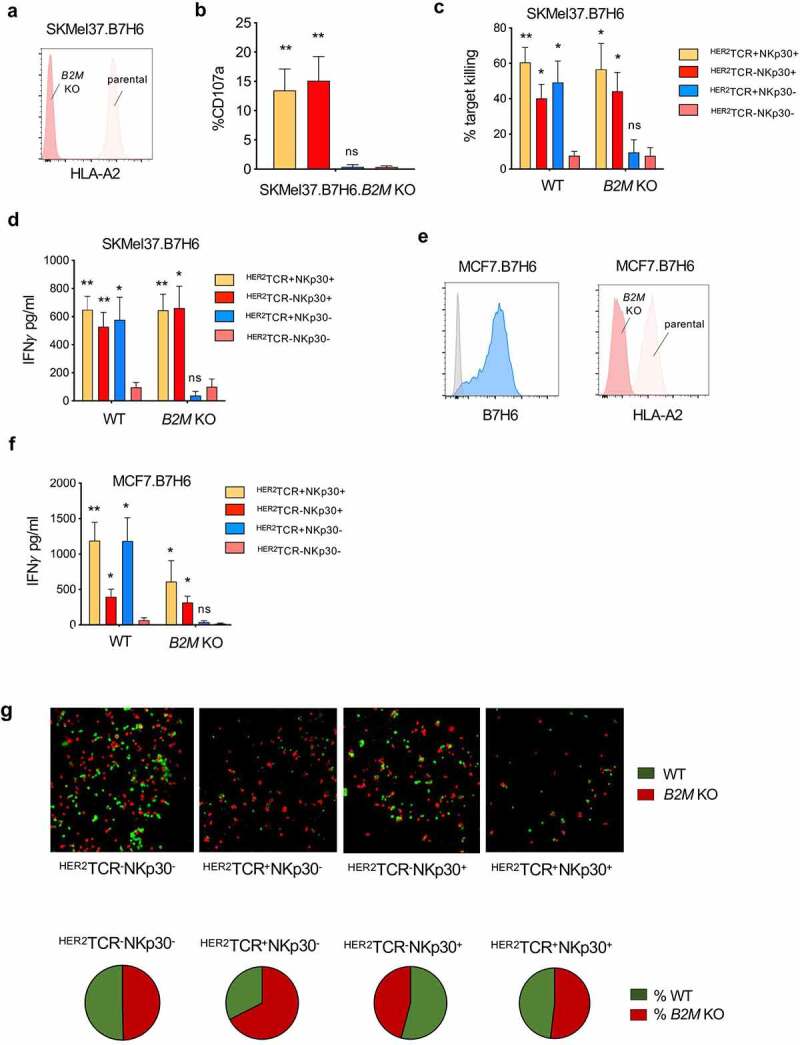
^HER2^TCR^+^NKp30^+^CD8^+^ T cells can respond to tumor cells both in an MHC-class I-restricted and unrestricted manner. (a) Histogram showing HLA-A2 expression on the surface of parental and *B2M* KO SKMel37.B7H6 cells (isotype control in gray). (b) Graph showing degranulation (CD107a^+^) on gated effector populations after co-culture with SKMel37.B7H6 *B2M* knockout cells, for 4 h in the presence of anti-CD107a antibodies. (c) Percentage of target killing and (d) IFN-*γ* production after 24 h of co-culture of the indicated FACS-sorted effector populations with the parental and *B2M* KO target cells. (e) Histograms showing B7H6 expression on the surface of parental MCF7.B7H6 cells (isotype control in gray) and HLA-A2 expression on parental and *B2M* KO MCF7.B7H6 cells. (f) IFN-γ production after 24 h co-culture of the indicated FACS-sorted effector populations with parental and *B2M* KO cells. n > 3 independent experiments, mean±SEM. *P < .05, **P ≤ 0.01, ns, not significant, unpaired Students *t*-test (comparison with ^HER2^TCR^−^NKp30^−^ T cell population). (g) SKMel37.B7H6 MHC-class I negative (red) and MHC-class I positive (green) cells were pre-labeled with cytoplasmic labeling dyes (CytoLight rapid reagents, Incucyte®) and mixed at a 1:1 ratio in microscopy chamber wells. Effector cells were added at a 3:1 ratio and the cells were co-cultured for 24 h at 37ºC. Pie-charts indicate % of red or green cells remaining after co-culture with the different effector cells. Images were acquired with a Leica SP5 microscope. Representative fields were selected from a combined full-well image obtained with a tile-scan using Leica software. Images were analyzed using ImageJ/Fiji software

MHC-class I downregulation is a well-established common evasion mechanism by tumor cells, allowing escape from T cell recognition. Accordingly, we performed a CRISPR/Cas9-mediated knockout of *B2M* in the SKMel37.B7H6 cell line, resulting in the loss of MHC-class I cell surface expression (Figure 4(a)). As shown in Figure 4(b), conventional ^HER2^TCR-bearing CD8^+^ T cells were not able to respond to targets lacking MHC-class I at the cell surface, while ^HER2^TCR^+^NKp30^+^CD8^+^ T cells degranulated similarly to NKp30^+^CD8^+^ T cells lacking an HER2-specific TCR (Figure 4(b)). Moreover, we evaluated the killing capacity of the different effector T cell subsets in response to tumor targets expressing or lacking MHC-class I at the cell surface. We observed that NKp30^+^CD8^+^ T cells effectively killed the tumor cells regardless of the expression of MHC-class I at the cell surface, while ^HER2^TCR^+^NKp30^−^CD8^+^ T cells could only kill tumor cells expressing MHC-class I (Figure 4(c)). Similar results were observed when measuring IFN-γ release by effector cells after co-culture with tumor cell targets. Conventional ^HER2^TCR^+^NKp30^−^CD8^+^ T cells produced IFN-γ only upon co-culture with MHC-class I-expressing cells, while ^HER2^TCR^+^NKp30^+^CD8^+^ T cells showed an increased IFN-γ response even in the absence of MHC-class I/peptide recognition (Figure 4(d)). NKp30^−^CD8^+^ T cells lacking the HER2-specific TCR could not respond or kill the cell lines regardless of the presence or absence of MHC-class I (Figure 4c and d). Similar results were observed when breast cancer cells overexpressing B7H6 (MCF7.B7H6), in which MHC-class I expression was abrogated by CRISPR/Cas9-mediated knockout of *B2M* (MCF7.B7H6.*B2M* KO) (Figure 4(e-f)), were used as targets.

Furthermore, we used additional target tumor cell lines expressing HER2, but lacking HLA-A2, the MHC-class I haplotype recognized by the transduced HER2-specific TCR. SKMel28.B7H6 melanoma cells and HeLa cells, a cervical carcinoma cell line endogenously expressing B7H6, were co-cultured with the CD8^+^ T cells (Figure S3a). Concordantly, NKp30^+^CD8^+^ T cells, carrying or not HER2-specific TCRs, could recognize and kill both tumor cell lines, while ^HER2^TCR^+^NKp30^−^CD8^+^ T cells were unable to kill or produce IFN-γ in response to HLA-A2-negative targets, regardless of ^HER2^TCR expression (Figure S3b-c).

In conclusion, our results indicate that innate-like NKp30^+^CD8^+^ T cells are able to display dual-response capacity, recognizing target cells based on both an antigen-specific recognition and an NK-like, TCR-independent manner, allowing killing of target cells that lack MHC-class I at the cell surface, but express cognate NK cell ligands.

### Targeting of tumor MHC-class I expression heterogeneity by ^HER2^TCR^+^NKp30^+^CD8^+^ T cells

Tumor cell immune escape based on MHC-class I downregulation leads to a heterogeneous tumor with cells expressing or lacking MHC-class I at the cell surface within same tissue. In order to address the killing of tumor cells in a MHC-class I positive/negative tumor target mixed setting, we established a fluorescence microscopy-based killing assay. MHC-class I-positive targets were labeled in green and MHC-class I-negative targets in red, mixed at a 1:1 ratio, and then co-cultured with the different effectors. We observed that ^HER2^TCR^+^NKp30^+^CD8^+^ T cells efficiently killed both MHC-class I^+^ targets (green) as well as targets lacking MHC-class I at the cell surface (red), contrarily to ^HER2^TCR^+^NKp30^−^CD8^+^ T cells (Figure 4(g)). NKp30^+^CD8^+^ T cells not harboring a HER2-specific TCR also efficiently killed target cells regardless of MHC-class I expression at the cell surface (Figure 4(g)). Noteworthy, while target co-culture with the subsets expressing NKp30 resulted in a similar percentage of remaining of viable MHC-class I^+^ or MHC-class I^−^ target cells, when ^HER2^TCR^+^NKp30^−^CD8^+^T cells were used as effectors, we observed an enriched fraction of viable MHC-class I-negative targets (red) remaining at the end of the co-culture period (Figure 4(g), pie chart). These results indicate that ^HER2^TCR^+^NKp30^+^CD8^+^ T cells are empowered to target heterogeneous tumors regardless of MHC-class I expression at the cells surface.

### Dual recognition modes of NKp30^+^CD8^+^ T cells carrying a tumor-reactive ^HER2^CAR

**Figure 5.**
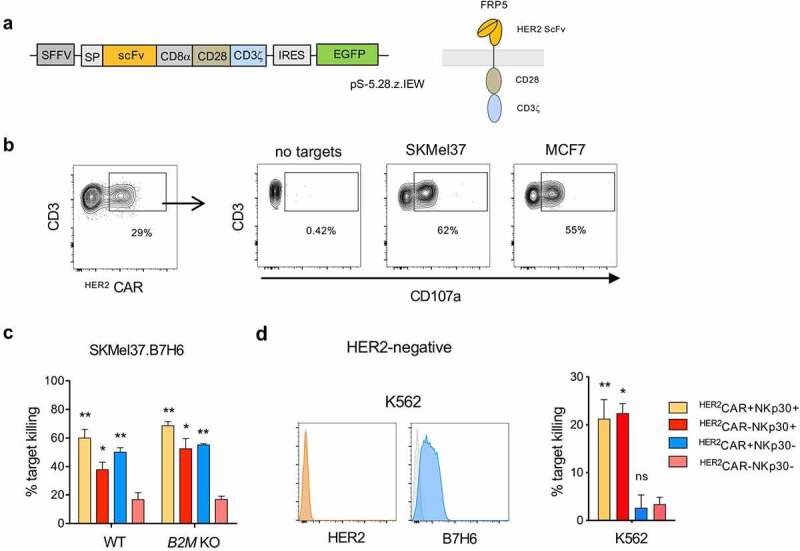
^HER2^CAR^+^NKp30^+^CD8^+^ T cells can eliminate tumor cells expressing or lacking HER2 using dual recognition modes. CD8^+^ T cells were expanded in the presence of αCD3/CD28 and IL-15 for 3 days, transduced with lentiviral particles encoding the HER2-CAR and then cultured with IL-15 for 12 more days. (a) Simplified schematic representation of the CAR construct with an extracellular HER2-specific scFv antibody domain, a CD8α hinge region, transmembrane and intracellular domains of CD28 and the intracellular domain of CD3ζ, followed by an internal ribosome entry site (IRES) and an enhanced green fluorescent protein sequence (EGFP). (b) Expression of the ^HER2^CAR on CD8^+^ T cells upon transduction based on GFP expression (left). Dot-plots showing degranulation after co-culture with HER2-expressing target cells (right). (c) Percentage of target killing after 24 h of co-culture of the indicated effector populations with parental SKMel37.B7H6 parental and *B2M* KO target cells. (d) Histograms showing HER2 and B7H6 expression on the surface of K562 cells (isotype control in gray) (left). Percentage of target killing after 24 h of co-culture of the indicated effector populations with K562 cells (right). n > 3 independent experiments, mean±SEM. *P < .05, **P ≤ 0.01, ns, not significant, unpaired students *t*-test (comparison with ^HER2^CAR^−^NKp30^−^ T cell population)

Besides arming T cells with specific TCRs, chimeric antigen receptor (CAR) transduction is a common strategy to retarget T cells to tumor-associated antigens. In order to generate ^HER2^CAR-expressing NKp30^+^CD8^+^ T cells, we established the lentiviral transduction of an HER2-targeting CAR into NKp30^+^CD8^+^ T cells. We used a humanized CAR based on the HER2 specific antibody, FRP5, and CD28 and CD3ζ signaling domains (CAR 5.28.z) (Figure 5(a)).^30^ We transduced both conventional CD8^+^ T cells and the NKp30^+^CD8^+^ T cell population with the HER2-targeting CAR (^HER2^CAR) (Figure S4a-b) and assessed their functional response. Upon co-culture with different HER2^+^ tumor cell lines, we observed degranulation of ^HER2^CAR-transduced T cells (Figure 5(b)), showing that HER2-targeting CAR-T cells were functional.

In order to assess their cytotoxic capacity, we isolated the four paired subpopulations by FACS cell sorting, NKp30^+^ or NKp30^−^ CD8^+^ T cells harboring or not the HER2-specific CARs, and co-cultured them with different target cell lines expressing HER2 on the cell surface. ^HER2^CAR-armed CD8^+^ T cells efficiently recognized and killed the tumor cells, even in the absence of MHC-class I, and regardless of the HLA-class I haplotype (Figure 5(c) and Figure S4c). As expected, contrarily to ^HER2^CAR^−^NKp30^−^CD8^+^ T cells, ^HER2^CAR^−^NKp30^+^CD8^+^ T cells could kill target tumor cells, indicative of the additional NK-like killing capacity of this population (Figure 5(c)). Interestingly, by using a HER2-negative B7H6-expressing target cell line (K562), we could show that ^HER2^CAR^+^NKp30^+^CD8^+^ T cells could effectively kill HER2-negative target cells contrarily to conventional ^HER2^CAR^+^NKp30^−^CD8^+^ T cells, which were unable to recognize those tumor targets (Figure 5(d)). These results demonstrate the dual-recognition capacity of ^HER2^CAR^+^NKp30^+^CD8^+^ T cells, empowered with a capacity to kill target cells even in the absence of HER2 expression.

## Discussion

Although the immune system is classically divided into innate and adaptive immunity, this conceptual stratification has increasingly blurred in recent years, with accumulating evidence that NK cells can acquire adaptive features, as immunological memory. Classically, T cells recognize tumor cells based on the expression of specific tumor-associated peptides in the context of MHC-class I molecules, while NK cells recognize and kill tumors based on an array of innate germline-encoded NK receptors expressed at the cell surface. NKp30 has been regarded as a *bona fide* marker of NK cells. However, recently, we have uncovered a population of CD8^+^ T cells expressing NKp30 and displaying broad innate features.^23^ Besides existing in a small percentage in circulating blood, we showed that IL-15 can *de novo* induce NKp30 to be expressed on CD8^+^ T cells, leading not only to the expansion of the preexisting NKp30^+^CD8^+^ T cell population, but to *de novo* generation of more NKp30^+^CD8^+^ T cells. Moreover, this IL-15-driven induction of NKp30 is accompanied by *de novo* expression of FcεRIγ, a signaling adaptor crucial for NKp30 function on this population. In 2009, B7H6 was discovered as a tumor-specific ligand for NKp30.^8^ In fact, B7H6 has been found to be upregulated in several tumor types, establishing the NKp30/B7H6 as an attractive axis to be exploited for immunotherapy,^9–13,31,32^ including the development of targeting approaches against B7H6.^31,33^

The adoptive transfer of T cells transduced with tumor antigen-specific TCRs or CARs has been increasingly appreciated as a promising immunotherapeutic strategy for cancer treatment. One of the major impairments of targeted adoptive cell therapies is tumor escape based on common immune evasion strategies, such as MHC-class I downregulation or reduced antigen expression. Indeed, MHC-class I downregulation has been described in 40–90% of tumors, frequently correlating with a worse prognosis.^34^ MHC-class I loss or downregulation can result from genetic, epigenetic, transcriptional, and post-transcriptional alterations, namely in the class I heavy chain genes, in β2M, and in the TAP-encoding genes.^34,35^ MHC-class I expression decrease and antigen loss lead to tumor cell invisibility to T cell-mediated killing, making engineered T cells incapable to recognize tumor cells.^2,34,36^ This causes outgrowth of tumor cells lacking MHC-class I and/or antigen, thus resulting in tumor editing and escape from adoptive T cell transfer therapy.^2,16^ Although several immunotherapeutic strategies have been developed to target HER2, the intratumoral heterogenous expression of HER2 has been reported to impair their therapeutic efficacy.^37,38^

Here, we successfully equipped NKp30^+^CD8^+^ T cells with specificity against HER2, to combine innate and specific adaptive recognition against tumor targets. ^HER2^CAR/ ^HER2^TCR expression was stably maintained on CD8^+^ T cells even after more than 60 days upon cell transduction (Figure S5). We could show that ^HER2^TCR^+^NKp30^+^CD8^+^ T cells recognize and kill tumor cells both in an MHC-dependent and MHC-independent manner, targeting intratumoral heterogeneity. Targeted innate-like NKp30^+^CD8^+^ T cells showed capacity to recognize and eliminate tumors in a ^HER2^TCR-dependent manner, similar to conventional ^HER2^TCR^+^CD8^+^ T cells, while in addition, exhibiting the distinctive capacity of recognizing targets in a NK-like manner when MHC-class I expression was lacking. Indeed, this was of particular advantage in targeting heterogeneous tumors composed of MHC-class I^+^ and MHC-class I^−^ tumor cells, where ^HER2^TCR^+^NKp30^+^CD8^+^ T cells showed the capacity to eliminate tumor cells even in the absence of MHC-class I and antigen display. Moreover, low MHC-class I expression and expression of NK ligands have been described for some cancer stem cells,^39,40^ suggesting an advantage of innate-like NKp30^+^CD8^+^ T cells to target cancer initiating cells that became invisible to T cell recognition.

CAR-T cell-based approaches target tumor antigens directly, without the need of MHC-peptide recognition. Here, we used a second-generation HER2-targeting CAR consisting of an HER2-specific scFv antibody domain fused via a linker to a composite CD28-CD3ζ signaling domain. We generated ^HER2^CAR^+^NKp30^+^CD8^+^ T cells exhibiting the capacity to target both HER2-expressing tumor cells, as well as cells lacking the specific antigen, using NK-like recognition. Our in silico analyses of TCGA datasets showed variable HER2 and B7H6 expression among cancer patients, and importantly, their intratumoral heterogeneous expression within the tumor tissue of the same patient. The remarkable ability of ^HER2^CAR^+^NKp30^+^CD8^+^ T cells to kill and recognize tumor cells not expressing the targeted tumor antigen using their dual-killing capacity, empowers this population against tumor escape based on antigen loss.

Here we show that NKp30^+^CD8^+^ T cells can be armed with tumor-specific TCRs or CARs targeting a tumor-associated antigen, increasing the spectrum of target cells that can be killed, independently of MHC-class I or antigen display. Noteworthy, as we have previously described, this NKp30^+^CD8^+^ T cell population shows broad NK-like properties.^23^ Indeed, besides NKp30 expression, these cells co-express other NK receptors, as well as higher amounts of cytolytic molecules and downstream signaling modules involved in the NK killing machinery. In addition, innate features of NKp30^+^CD8^+^ T cells are accompanied by reduced expression of checkpoint inhibitor molecules,^23^ indicating that these cells might be less susceptible to inhibitory circuits in the tumor microenvironment.^23^ This NKp30^+^CD8^+^ T cell population is unlike other innate-like T cells previously described as dependent on HLA-E recognition.^41–43^ Indeed, we could show that NKp30^+^CD8^+^ T cells recognize and kill target cells deficient in B2M, which do not display HLA-E at the cell surface. Thus, this unique effector population can be further exploited for immunotherapy by its capacity to target different tumor cell ligands beyond the NKp30-B7H6 interaction, broadening even further their potential to target tumor cell heterogeneity.

Besides their dual-recognition potential, we have previously described that under weak TCR signal triggering, co-stimulation with NKp30 can lead to a synergistic response. Thus, harnessed NKp30^+^CD8^+^ T cells might exert superior effector potential to targets with low expression of MHC-class I. Moreover, the fact that NKp30^+^CD8^+^ T cell population also co-express inhibitory NK receptors, such as KIRs,^23^ increases the specificity toward tumor cells and may enhance their safety for adoptive transfer therapies.

The unique capacity of eliminating tumor cells with NK-like recognition and combined TCR/CAR-specificity appears as a promising cell-based immunotherapy strategy targeting tumor heterogeneity and circumventing common immune evasion strategies by tumor cells.

## Material and methods

### Transcript and protein expression data of HER2 and B7H6 from cancer patients

Transcript expression data of *ERBB2* and *NCR3LG1* from several tumors from TCGA data sets (PanCancer Atlas) were extracted from the cBioPortal for Cancer Genomics (https://cbioportal.org). Graphs and correlations were generated in the cBioPortal software. Immunohistochemistry of tumor tissue slides of paired HER2 and B7H6 protein expression in breast cancer and colon carcinoma from patient samples was originally obtained from the Human Protein Atlas database (http://www.proteinatlas.org/).^44^ Tissue microarrays were stained with antibodies and labeled with DAB (3,3ʹ-diaminobenzidine) and counterstained with hematoxylin. Each sample is represented by 1 mm tissue cores. In the Human Protein Atlas database, all images of tissues stained by immunohistochemistry are manually annotated by two specialists sequentially. Annotation parameters include an evaluation of i) staining intensity (negative, weak, moderate or strong) and ii) fraction of stained cells within a sample (<25%, 25–75% or >75%). The staining intensity is classified as negative, weak, moderate or strong based on the laser power and detector gain settings used for image acquisition in combination with the visual appearance of the image.

### Cell isolation and culture

Buffy coats from healthy donors were obtained from DRK-Blutspendedienst Baden-Württemberg-Hessen (Mannheim, Germany). Written informed consent from the blood donors was obtained and ethical approval 87/04 was granted by the Ethik Kommission II of the Medical Faculty Mannheim (Mannheim, Germany). Peripheral blood mononuclear cells (PBMCs) were isolated by gradient centrifugation (Biocoll Separating Solution, Biochrom). CD8^+^CD56^−^ T cells were purified using first a Human CD8^+^ T Cell Isolation Kit (Miltenyi Biotec), followed by a further isolation step using CD8 MicroBeads (both Miltenyi Biotec). Purified CD8^+^ T cells were cultured in RPMI 1640 medium (Sigma-Aldrich) with 10% human serum (PAA), 1% Penicillin/ Streptomycin and 10 ng/ml of recombinant human IL-15 (R&D Systems), as previously described.^23^

### Antibodies

The following antibodies were used: anti-human CD3 (HIT3a, Biolegend), anti-human CD8 (SK1, Biolegend), anti-human NKp30 (P30-15, Biolegend), anti-human HLA-A2 (BB7.2, Biolegend), anti-human HER2 (24D2, Biolegend), anti-mouse TCR-β chain (H57-597, Biolegend), anti-human CD107a (H4A3, Biolegend), anti-human IFN-γ (B27, Biolegend). Corresponding isotype controls mIgG1 (MOPC-21, Biolegend), mIgG2b (MG2b-57, Biolegend) and Armenian Hamster IgG (HTK888, Biolegend) were used. Anti-B7H6 clone 1.18 and corresponding isotype control were from in-house production.^9^ For CD8^+^ T cell activation, anti-human functional-grade purified anti-CD3 (UCHT1, Biolegend) and anti-CD28 (37.51, Biolegend) were used.

### Flow cytometry analysis and FACS sorting

For surface staining, cells were incubated with antibodies in staining buffer for 30 min at 4°C. Cells were acquired using a FACS Fortessa™ (BD) and data were analyzed using FlowJo software. For FACS-sorting after transduction with HER2-specific TCRs or CARs, CD8^+^ T cells were washed twice and stained for 30 min at 4°C in PBS. Viable T cells (CD3^+^7AAD^−^) were gated according to NKp30, mouse TCR-β (for TCR-transduced cells) or GFP (for CAR-transduced cells) expression. Cells were sorted using a FACSAria™ Fusion cell sorting instrument (BD). Sorted cells were left in culture overnight before being used for functional assays.

### Cell lines

The cell lines MCF7, SKMel37, HeLa and HEK293T were cultured in D-MEM (Sigma-Aldrich) supplemented with 10% FCS and 1% Penicillin/Streptomycin. K562 were cultured in RMPI (Sigma-Aldrich) supplemented with 10% FCS and 1% Penicillin/Streptomycin. Cells were dissociated with trypsin-EDTA (Sigma-Aldrich) for regular splitting or with non-enzymatic dissociation solution (Sigma-Aldrich) for analysis of ligands expression and functional assays. All cell lines were authenticated.

### *B7H6 overexpression and CRISPR/Cas9* B2M *knockout in cell lines*

For B7H6 overexpression, cell lines were retrovirally transduced with pMXneo-B7H6 as previously described.^23^ For *B2M* CRISPR/Cas9 knockout, B2M sgRNA CRISPR/Cas9 All-in-One Lentivector (Human) plasmids were used (ABM, Richmond, BC, Canada). Packaging and envelope plasmids pMD2.G and psPAX2 were a gift from Didier Trono (Addgene plasmids #12259 and #12260).

### Functional assays

For degranulation assays, anti-CD107a mAb was added to the co-cultures of tumor cells and effector cells. After 1 h of incubation, GolgiStop™ (BD) was added and incubated for four additional hours at 37°C. For *in vitro* cytotoxicity studies, FACS-sorted CD8^+^ T cells (NKp30^+^ or NKp30^−^ bearing or not ^HER2^TCR or ^HER2^CAR) from the same donor were co-cultured with the indicated cell lines for 24 h at 37°C. Target cell killing was determined by using the LDH-Glo™ (Promega) assay kit. Target cells were lysed for maximum release determination. % specific lysis = 100× ((LDH release)-(spontaneous LDH release))/((maximum LDH release)-(spontaneous LDH release)). For functional assays, cells were cultured in RPMI 1640 medium (Sigma-Aldrich) with 10% human serum (PAA), 1% Penicillin/Streptomycin and recombinant human IL-15 (R&D Systems), as above described.

For determination of IFN-γ production, supernatants from effector and target cell co-cultures were collected and cytokine concentration was quantified using ELISA MAX™ Deluxe Set for Human IFN-γ (Biolegend), according to the manufacturer's instructions.

### Fluorescence microscopy

SKMel37.B7H6 cells were incubated with IncuCyte® CytoLight Rapid Green or Red Reagent (Essen Bioscience), washed and plated in microscopy Nunc™ Lab-Tek™ Chamber Slides (Thermo Fisher Scientific) to adhere. Afterward, medium was removed, and effector cells were added in RPMI medium without phenol red (Thermo Fisher Scientific) and the co-cultures were incubated for 24 h at 37°C. Images were acquired with a LEICA TCS SP5 DS (Leica) confocal microscope and analyzed with the Fiji software (http://fiji.sc/Fiji).

### Generation of HER2-TCR and HER2-CAR viral particles and CD8^+^ T cell transduction

For generation of HER2-specific TCR transduced CD8^+^ T cells, a construct with P2A-linked TCRα- and β-chain genes, murinized, codon-optimized, and inserted as a single transgene cassette into the retroviral vector MP71-PRE, was used (kindly provided by Prof Dr Wolfgang Uckert, MDC, Berlin). Packaging plasmids gag/pol (pcDNA3.1-MLVg/p) and env (pALF-MLV-10A1) were used.^29^ For generation of HER2-specific CAR-T cells, a construct with a humanized CAR based on HER2-specific antibody, FRP5, harboring CD28 transmembrane and intracellular domains, and CD3ζ intracellular domain (CAR 5.28.z), was introduced into a lentiviral transfer plasmid, generating a pS-5.28.z-IEW construct expressing GFP, as previously described.^30^ Packing and envelope plasmids pMD2.G and psPAX2 were a gift from Didier Trono (Addgene plasmids # 12259# 12260). Viral supernatants were obtained after transfection of HEK293T cells using lipofectamine (Sigma-Aldrich), filtered through 0.45 µm PVDF syringe filters (Millipore), and concentrated using Retro-Concentin Virus Precipitation Solution (System Biosciences). CD8^+^ T cells pre-activated with αCD3/αCD28 plate-bound antibodies and 25 ng/ml IL-15 for 3 days were transduced by spinoculation with the viral supernatants, as previously described.^23^ Transduced cells were then expanded for additional 10 days in 10 ng/ml IL-15 and cell subpopulations were FACS-sorted for functional assays.

### Statistics

Student’s *t*-test was used to compare each population against the control population NKp30^−^CAR^-^/ TCR^−^. *P* < .05 was considered significant. All tests are indicated in each corresponding individual figure legend.

## Supplementary Material

Supplemental Material
