# Supplementary material for: Innate-like NKp30+CD8+ T cells armed with TCR/CAR target tumor heterogeneity
Source: Oncoimmunology. 2021 Oct 19;10(1):1973783. doi: 10.1080/2162402X.2021.1973783 (PMC8758178; doi:10.1080/2162402X.2021.1973783)
Supplement: Supplemental Material [file KONI_A_1973783_SM4927.pdf]

Supplementary Information for

Innate-like NKp30^{+}CD8^{+} T cells armed with TCR/CAR target tumor heterogeneity

Authors: Margareta P. Correia, Ana Stojanovic, Winfried Wels, Adelheid Cerwenka

Supplementary Fig. S1

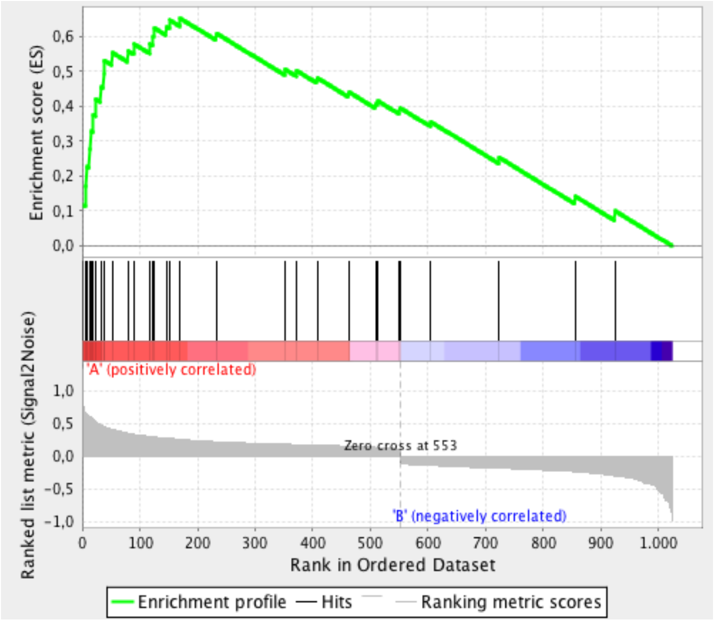

Fig. S1. NKp30^{+}CD8^{+} T cell population displays broad NK-like features. Purified CD8+ T cells were FACS-sorted into NKp30^{+} and NKp30^{-} CD8^{+} T cell populations after 12 days culture with IL-15, and whole-genome expression analysis was performed using Illumina arrays (n=6 independent donors). Differentially expressed genes between NKp30^{+} and NKp30^{-} CD8^{+} T cells (cut-off absolute log2-fold-change≥0.5) were analyzed in Gene set enrichment analysis (GSEA) [1, 2]. Enrichment Plot shows KEGG Natural Killer Cell Mediated Cytotoxicity enrichment profile for NKp30^{+} CD8^{+} T cells.

Supplementary Fig. S2

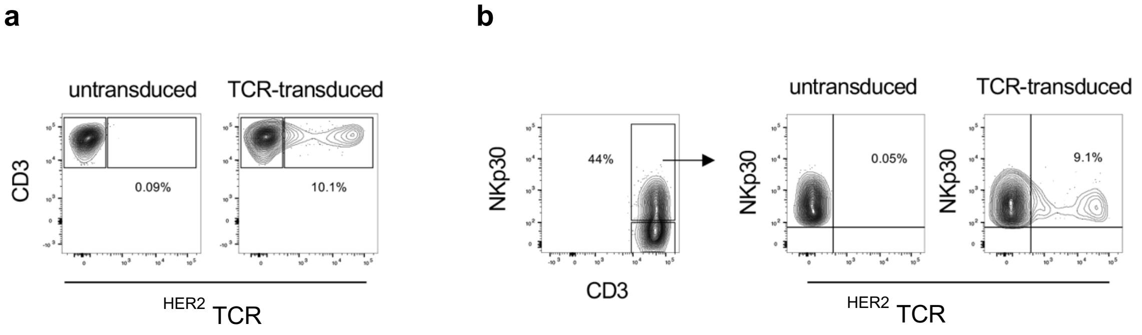

Fig. S2. Generation of NKp30$^+$CD8$^+$ T cells harboring HER2-specific TCRs. CD8$^+$T cells transduced with retroviral particles encoding HER2-specific TCRs (^{HER2}TCR). **(a)** Representative plot showing expression of ^{HER2}TCRs on bulk CD8$^+$ T cells upon transduction. **(b)** Contour-plot showing ectopic ^{HER2}TCR expression on pre-gated NKp30$^+$CD8$^+$ T cells upon transduction.

Supplementary Fig. S3

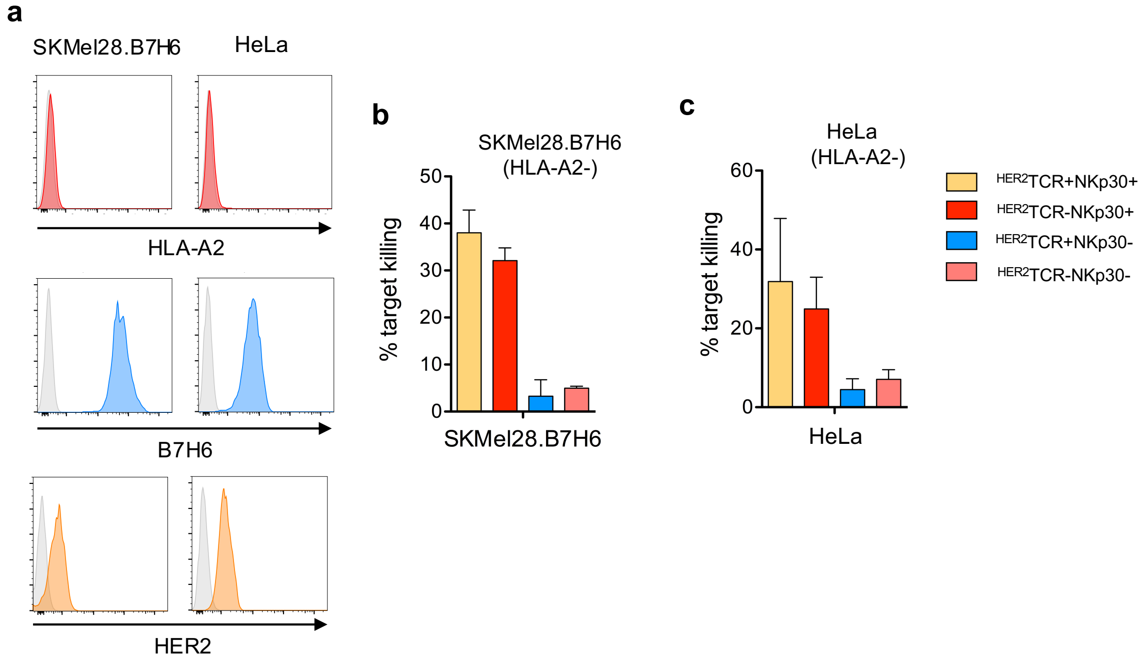

Fig. S3. ^{HER2}TCR^{+}NKp30^{+}CD8^{+} T cells recognize and kill MHC-mismatched tumor cells. (a) Cell lines expressing HER2, B7H6 and negative for HLA-A2 expression on the cell surface were selected. SKMel37.B7H6 (melanoma cell line), HeLa (cervical cancer cell line). **(b)** Percentage of target killing upon 24h co-culture with the indicated FACS-sorted ^{HER2}TCR^{+}NKp30^{+}, ^{HER2}TCR^{+}NKp30^{-}, ^{HER2}TCR^{-}NKp30^{+} or ^{HER2}TCR^{-}NKp30^{-} CD8+ T cells.

Supplementary Fig. S4

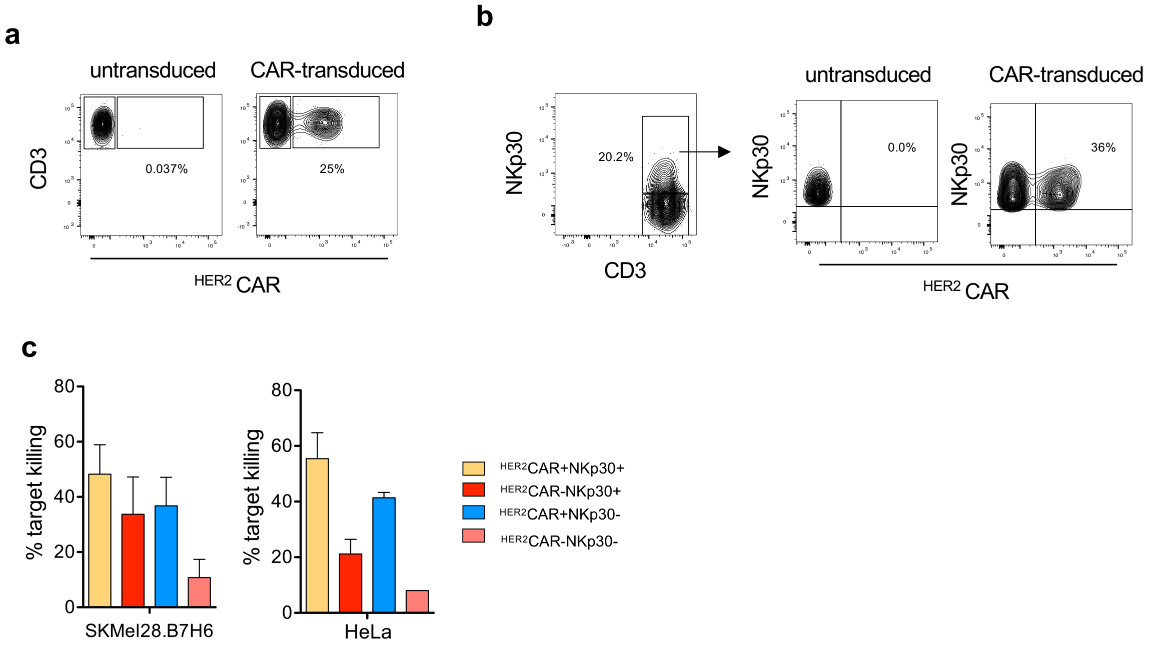

Fig. S4. Generation of NKp30$^+$CD8$^+$ T cells armed with HER2-specific CARs. CD8$^+$ T cells were transduced with lentiviral particles encoding HER2-directed CARs (^{HER2}CARs). **(a)** Representative plot showing expression of ^{HER2}CARs on bulk CD8$^+$ T cells upon lentiviral transduction. **(b)** Contour-plot showing ^{HER2}CAR expression on pre-gated NKp30$^+$CD8$^+$ T cells upon transduction. **(c)** Percentage of target killing upon 24h co-culture with the indicated FACS-sorted ^{HER2}CAR$^+$NKp30$^+$, ^{HER2}CAR$^+$NKp30$^-$, ^{HER2}CAR$^-$NKp30$^+$ or ^{HER2}CAR$^-$NKp30$^-$ CD8+ T cells.

Supplementary Fig. S5

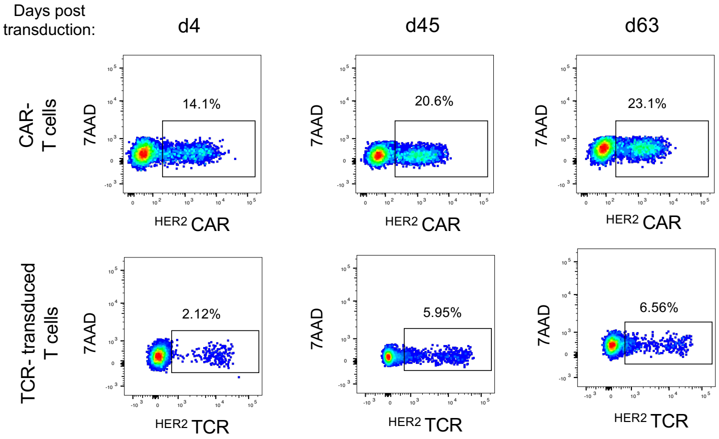

Fig. S5. HER2-specific TCRs and CARs ectopically expressed on CD8+ T cells are stably maintained over time. CD8$^+$ T cells transduced with retroviral particles encoding HER2-specific CARs or TCRs were maintained in IL-15-supplemented media. Representative plots showing expression of ^{HER2}TCRs/ ^{HER2}CARs on bulk CD8$^+$ T cells 4, 45 and 63 days upon transduction.

References

1. Mootha, V.K. *et al.* PGC-1alpha-responsive genes involved in oxidative phosphorylation are coordinately downregulated in human diabetes. *Nat Genet* **34**, 267-273 (2003).

2. Subramanian, A. *et al.* Gene set enrichment analysis: a knowledge-based approach for interpreting genome-wide expression profiles. *Proc Natl Acad Sci U S A* **102**, 15545-15550 (2005).
